# R/parallel – speeding up bioinformatics analysis with R

**DOI:** 10.1186/1471-2105-9-390

**Published:** 2008-09-22

**Authors:** Gonzalo Vera, Ritsert C Jansen, Remo L Suppi

**Affiliations:** 1 Groningen Bioinformatics Centre (GBiC), Groningen Biomolecular Sciences and Biotechnology Institute, University of Groningen, Haren, The Netherlands; 2 Computer Architecture and Operating Systems Department (CAOS), Universitat Autònoma de Barcelona, Bellaterra, Spain

## Abstract

**Background:**

R is the preferred tool for statistical analysis of many bioinformaticians due in part to the increasing number of freely available analytical methods. Such methods can be quickly reused and adapted to each particular experiment. However, in experiments where large amounts of data are generated, for example using high-throughput screening devices, the processing time required to analyze data is often quite long. A solution to reduce the processing time is the use of parallel computing technologies. Because R does not support parallel computations, several tools have been developed to enable such technologies. However, these tools require multiple modications to the way R programs are usually written or run. Although these tools can finally speed up the calculations, the time, skills and additional resources required to use them are an obstacle for most bioinformaticians.

**Results:**

We have designed and implemented an R add-on package, R/parallel, that extends R by adding user-friendly parallel computing capabilities. With R/parallel any bioinformatician can now easily automate the parallel execution of loops and benefit from the multicore processor power of today's desktop computers. Using a single and simple function, R/parallel can be integrated directly with other existing R packages. With no need to change the implemented algorithms, the processing time can be approximately reduced *N*-fold, *N *being the number of available processor cores.

**Conclusion:**

R/parallel saves bioinformaticians time in their daily tasks of analyzing experimental data. It achieves this objective on two fronts: first, by reducing development time of parallel programs by avoiding reimplementation of existing methods and second, by reducing processing time by speeding up computations on current desktop computers. Future work is focused on extending the envelope of R/parallel by interconnecting and aggregating the power of several computers, both existing office computers and computing clusters.

## Background

In recent years, R [1] has gained a large user community in bioinformatics thanks to its simple but powerful data analysis language. Growing repositories like Bioconductor [2] and CRAN [3] assist bioinformaticians with hundreds of free analytical methods and tools. These user-contributed methods are easily reused and adapted to each particular experiment for analysis of biological data. Examples of often reused and adapted methods are, respectively, the packages tilingArray [4] and affyGG [5]. However, while data generated in experiments previously fitted on a CD-ROM, nowadays, using new equipments, hardly fit on a single DVD-ROM. As a consequence of the post-genomic explosion of data, the demand of computational power is increasing continuously and solutions to keep the *processing pace *of high-throughput devices are required. A common approach in many bioinformatics fields like genomics, transcriptomics and metabolomics, where large sequential data sets are analyzed, is the use of parallel computing technologies [6].

Using R together with parallel computing is not a trivial task as the language does not provide mechanisms to support it natively. To compensate for this lack, several tools have been developed with different degrees of success. Early contributions to parallel computing in R were based on available general purpose parallel computing frameworks like MPI [7] and PVM [8]. Examples of these R libraries are rmpi [9] and rpvm [10]. These libraries provide low level programming interfaces, the complexity of which hinders a wider use of them. In order to hide such complexity, packages like NetWorkSpaces [11], snow [12] or taskPR [13] were created. They provide a higher level of abstraction, encapsulating the previous libraries (i.e. rmpi, rpvm) in simpler libraries and providing sufficient flexibility for the average type of programs coded in R. Additional development has been carried out with the framework pR [14]. It adds several modules to automate the parallelization of any R program. This feature is very important since programmers do not need to think *"in parallel" *when coding their R scripts, and anyone without previous knowledge of parallel computing can benefit from its advantages. However, while the programming model has been simplified during the last years, the dependency on external frameworks and dedicated resources is still a major obstacle for many bioinformaticians (e.g. pR depends on a complex installation to access a cluster of MPI enabled servers). These solutions are well suited for research groups with access to dedicated infrastructures (e.g. computing clusters managed by skilled technicians) and/or enough time to invest in the development of *ad hoc *parallel programs. However, when these requirements are not met, solutions based on self-contained tools (e.g. squid for Perl [15]), capable of running in common desktop computers, are the preferred choice.

In this paper we present an R add-on package for parallel computing: R/parallel. To use it, the programmer does not need to change his algorithm nor install and maintain any additional software as the R/parallel package is self-contained and capable of using current multicore processor desktop computers. It easily and effectively enables the automatic parallelization of loops without data dependencies [16], thus bringing the benefits of parallel computing within the reach of any bioinformatician using R. Its design allows its direct use with current bioinformatics analysis tools such as R/qtl [17], MetaNetwork [18] or affyGG [5] for analysis of genome-wide gene expression data.

## Implementation

The implementation of R/parallel has been carried out with the objective of increasing the performance of R by means of parallel computing while minimizing the requirements to use it. This section explains the design decisions made to speed up R programs while overcoming the common problems experienced by bioinformaticians with previous parallel computing solutions.

The first aspect taken into account is the desire to minimize user intervention when parallelizing new or existing R programs. The perfect solution should not require any further modification from the programmer. This is achieved with fully automatic parallelizers, which parse the program code, check it for data dependencies and generate a set of independent tasks that can be safely evaluated in separate processors. However, the drawback of this approach is that the parallelizer, *a priori*, does not know the execution time of each independent task. When a set of tasks are running concurrently, additional overhead and delays are introduced due to additional processing steps (e.g. code replication or task coordination). It is quite likely that a sequence of small fast tasks is parallelized and, despite parallel execution, as a result of the transformation process and additional synchronization, the overall processing time can be increased. To avoid this situation, the design decision made is to let the users indicate which code regions (i.e. loops) they need to speed up. With this information, R/parallel will automatically parallelize its execution to increase the performance.

Another aspect to consider when developing parallel programs is the difficult task of debugging when coding errors arise. When multiple processing units are running concurrently at different steps of a program, the identification of the conditions that triggers a bug and the retrieval of the state of each execution thread is a cumbersome task that should be avoided. To minimize this risk, an objective of the design of this package is the ability to run the sequential (and parallel) version of the R programs without changing any further line of code. By running a program sequentially it is possible to test the correctness of the implemented algorithm and debug using traditional tools. The user can activate the parallel execution just by loading the R/parallel package before performing a calculation. This design decision is also supported by the fact that, as the user program is not functionally dependent on R/parallel, it can always be shared with other bioinformaticians without requiring them to install the package or modify a single line of code.

Internally, R/parallel is designed using a master-worker architecture. The master component runs within the main R instance and distributes the work. The workers run in new processes with new R instances and perform the calculations retrieved from the master. The implementation of the package uses R scripts and C++ objects, taking advantage of both programming worlds. Combining low level operating system calls in C++ to manage processes, threads and inter-process communications (IPC) with the intrinsic features of R, like the capability of retrieving or creating functions at runtime (a feature known as "computing on the language" [19]), it has been possible to build a generic solution able to automatically transform a sequential loop and parallelize its execution.

## Results

### Using the tool

**Figure 1:**
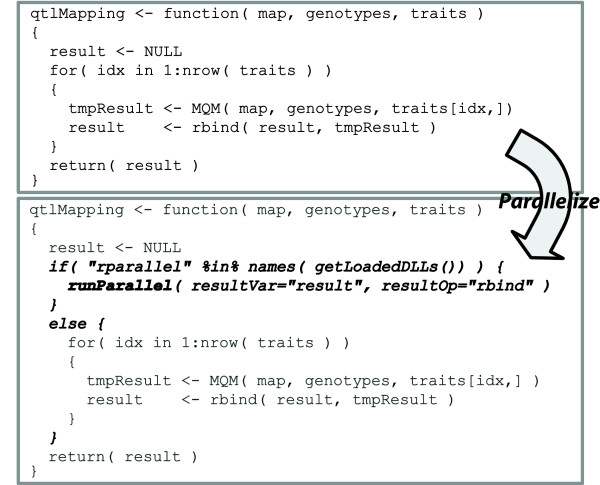
**Example using R/parallel**. To parallelize a loop it is only needed to add an if-else structure. The loop to be parallelized is placed inside the else body and the parallelizer function runParallel inside the if body. The last step is to indicate in runParallel the variable names used to accumulate the partial results and the operations to apply after each iteration. Other arguments like the number of parallel processes (workers) are optional. Detailed documentation and examples can be found on the project web page as well as within the package as R help pages.

Figure 1 shows with an example how easy it is to parallelize a loop in R with R/parallel. In this example, a long vector of gene expression data (i.e. traits) is analyzed through a loop to find quantitative trait loci (QTL) underlying variation in gene expression using a multiple QTL model (MQM) approach [20]. Once a programmer has finished coding and testing his function as usual, he only needs to add the lines shown (i.e. the runParallel function and the if-else conditional structure) to run it faster in parallel. Adding the lines explained, the execution time when processing 37685 traits from 73 individuals is reduced, using a quad-core processor, from approximate 4 hours to 1 hour. Another advantage of R/parallel is that it can be used in *batch mode *as well as in *interactive mode*. Moreover, to preserve functionality and allow code sharing, if R/parallel has not been installed and loaded, the if-else instructions added prevent parallelization from taking place and the loop will run sequentially.

### Use cases

**Figure 2:**
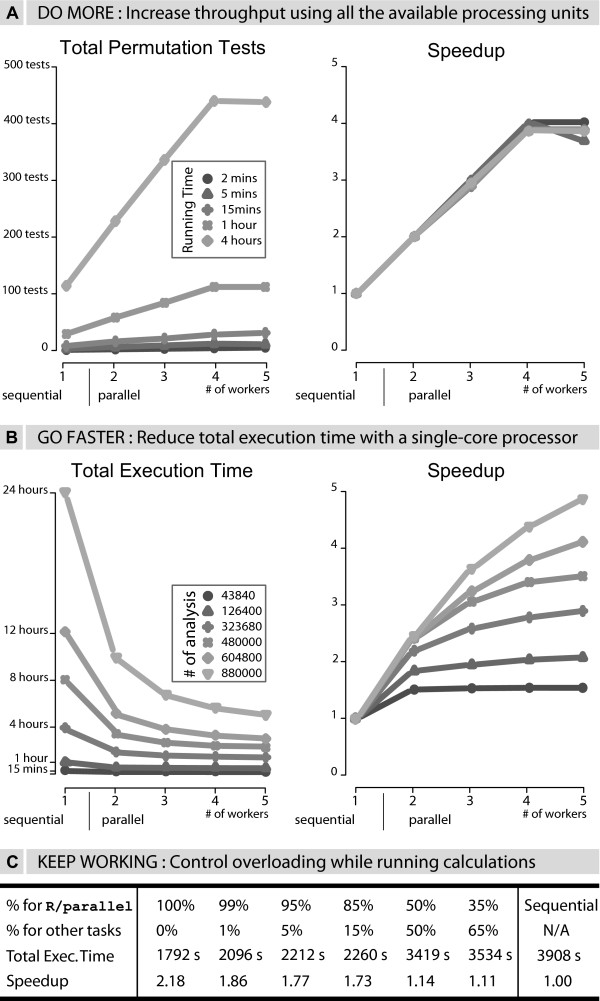
**Performance results**. **A) **The speedup increases linearly with the number of used cores. Setting more workers (5) than existing cores (4) does not improve the results. **B) **The (super linear) speedup exceeds the theoretical maximum of number of processing units due to faster tasks. **C) **With a conservative average load of 15% for other tasks, the computer is overloaded when R/parallel claims 100%. By reducing the percentage for R/parallel (with an optional argument) we can recover responsiveness and keep working on other tasks while running our calculations.

Practical applications of parallel computing are to increase the number of finished tasks given a fixed time or to decrease the time needed to perform a long task. To achieve this, a divide and conquer approach is used. The initial problem is partitioned into independent tasks which are computed simultaneously using several processing units. With R/parallel, partitioning is applied to loops and data, and multi-processing is used to get access to all the available processing units (i.e. *cores *in current desktop processors). The benefits of partitioning and multi-processing are shown in Figure 2 with three real cases. The observed speedups demonstrate that loops without data dependencies can be executed more efficiently using R/parallel. Obviously, with short calculations the speedup is minimal because of the additional overhead raised by the parallelization.

Typical bioinformatics cases where parallel computations are more often used are permutation tests or heuristic searches of multivariate spaces where, due to time constraints, the best result has to be computed before a deadline. The **case 2A **illustrates the increase of completed analyses (i.e permutation tests) by using all the available processing units. The function qtlThreshold.sma from the package affyGG is used with a quad-core processor to analyze a large number of permuted data sets using the same statistical analysis to compute (approximate) significance thresholds. Incrementing the number of parallel processes (i.e. workers) the usage of more cores has been enabled and therefore the overall performance has increased. The **case 2B **illustrates the decrease of the total execution time after parallelizing a problem. The function qtlMap.xProbe from the package affyGG is used with a single-core processor to compute the same statistical analysis over large data sets. In this case, due to the way memory is managed in R with linked lists and as a result of partitioning, small and faster tasks (with faster data indexing) are created. Therefore, in cases like this, even with a single processing unit, the total execution time is reduced. 

The **case 2C**, where the function qtlMap.xProbe is used this time with a dual-core processor, illustrates the problem of processor overloading and how to address it. An inconvenience when the processor load is 100% (i.e. the computer is overloaded) is that ready-to-run processes have to wait for the processor(s) to be run. This delay leads to downgraded response times in interactive programs that makes it hard, if not impossible, to keep working with the computer when simultaneously running intensive calculations. Fortunately, common office applications on today's desktop computers rarely claim more than 1% of the processor [21]. Therefore, by giving up a small percentage of the processor, it is possible to keep using the computer for other tasks, while the ongoing calculation only takes slightly more time.

## Conclusion

R/parallel, as shown, saves time to bioinformaticians in their daily tasks of analyzing experimental data. It effectively removes the most common obstacles encountered by bioinformaticians approaching parallel computing in R, like complex programming models or external dependencies on hard-to-maintain software frameworks. R/parallel is an easy-to-use R package which allows any programmer to parallelize their loops in a matter of minutes. The results demonstrate that R/parallel efficiently increases the performance of R when running parallel computations in current multicore processor desktop computer. As a consequence, bioinformaticians are able to approach reducing the processing time of a growing number of analytical methods by *N*-fold, *N *being the number of present cores in their computers.

Future work is focused on extending the functional and performance capabilities of R/parallel.

Additional functionalities like support for task parallelism or delayed loading of input data will extend the usability of the package. Additional performance, by expanding R/parallel from a single desktop computer to an office with several desktop computers or even a server farm, is our next milestone to speed up bioinformatics analysis with R.

We encourage any users to share their experiences with the authors to contribute to the extension of R/parallel.

## Availability and requirements

• **Project home page: **

• **Operating system(s): **Windows and Linux

• **Programming language: **R 2.6, C, C++

• **Other requirements: **none

• **License: **GPL for non-profit organizations

• **Any restrictions to use by non-academics: **licence needed

## Authors' contributions

GV conceived, designed and implemented the software. He wrote an early draft of the manuscript. RCJ provided end user requirements and practical examples to assess the usability of this tool. RLS provided direction and technical advise on the design and implementation. All three authors read, revised and approved the final manuscript.
